# Supplementary material for: Diverse signatures of convergent evolution in cactus-associated yeasts
Source: PLoS Biol. 2024 Sep 23;22(9):e3002832. doi: 10.1371/journal.pbio.3002832 (PMC11449361; doi:10.1371/journal.pbio.3002832)
Supplement: S1 Fig — The Saccharomycotina species tree phylogeny is represented with the respective branch labels as in Opulente and colleagues [51]. Cacti association is shown in the outer circle next to the respective species/strain (dark green–strictly cactophilic, light green–transient). The data underlying this Figure can be found in https://doi.org/10.6084/m9.figshare.24114381. (PDF) [file pbio.3002832.s001.pdf]

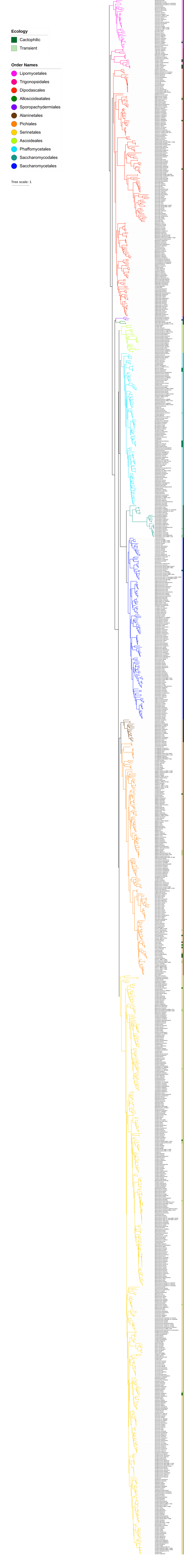

Ecology
Cactophilic
Transient
Order Names
Lipomycetales
Trigonopsidales
Dipodascales
Alloscoideatales
Sporopachydermiales
Alaninetales
Pichiales
Serinetales
Ascoideales
Phaffomycetales
Saccharomycodales
Saccharomycetales
Tree scale: 1
