# Supplementary figures and images for: Diverse signatures of convergent evolution in cactus-associated yeasts
Source: PLoS Biol. 2024 Sep 23;22(9):e3002832. doi: 10.1371/journal.pbio.3002832 (PMC11449361; doi:10.1371/journal.pbio.3002832)

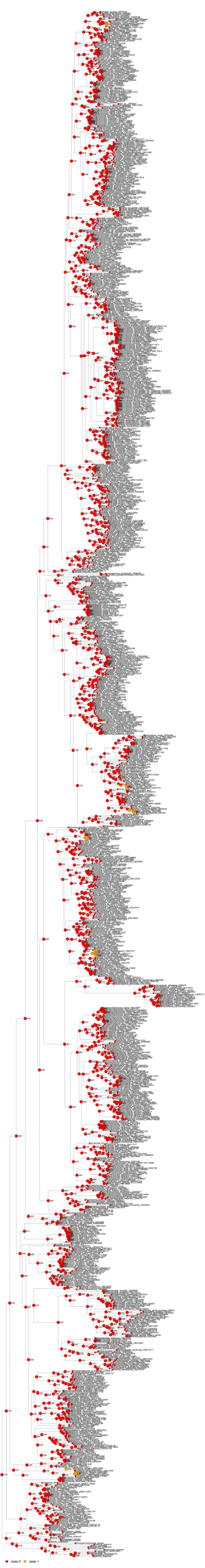

state 0 state 1

Supplement: S2 Fig — The number of independent events of cacti association were inferred by performing an ancestral state reconstruction using the MBASR toolkit [135] and the topology of the species tree presented in Fig 1. State 0 (red) means absence of the trait while State 1 (orange) means presence of the trait and were attributed according to S1 Table (considering presence of the trait only the strictly cactophilic species). For each node in the tree, probability of the trait being present (orange) or absent (red) is represented as a pie chart. In each terminal branch, red or orange circles represent the extant state (used as an input) for each species. Numbers next to each node represent the node identification. (PDF) [file pbio.3002832.s002.pdf]

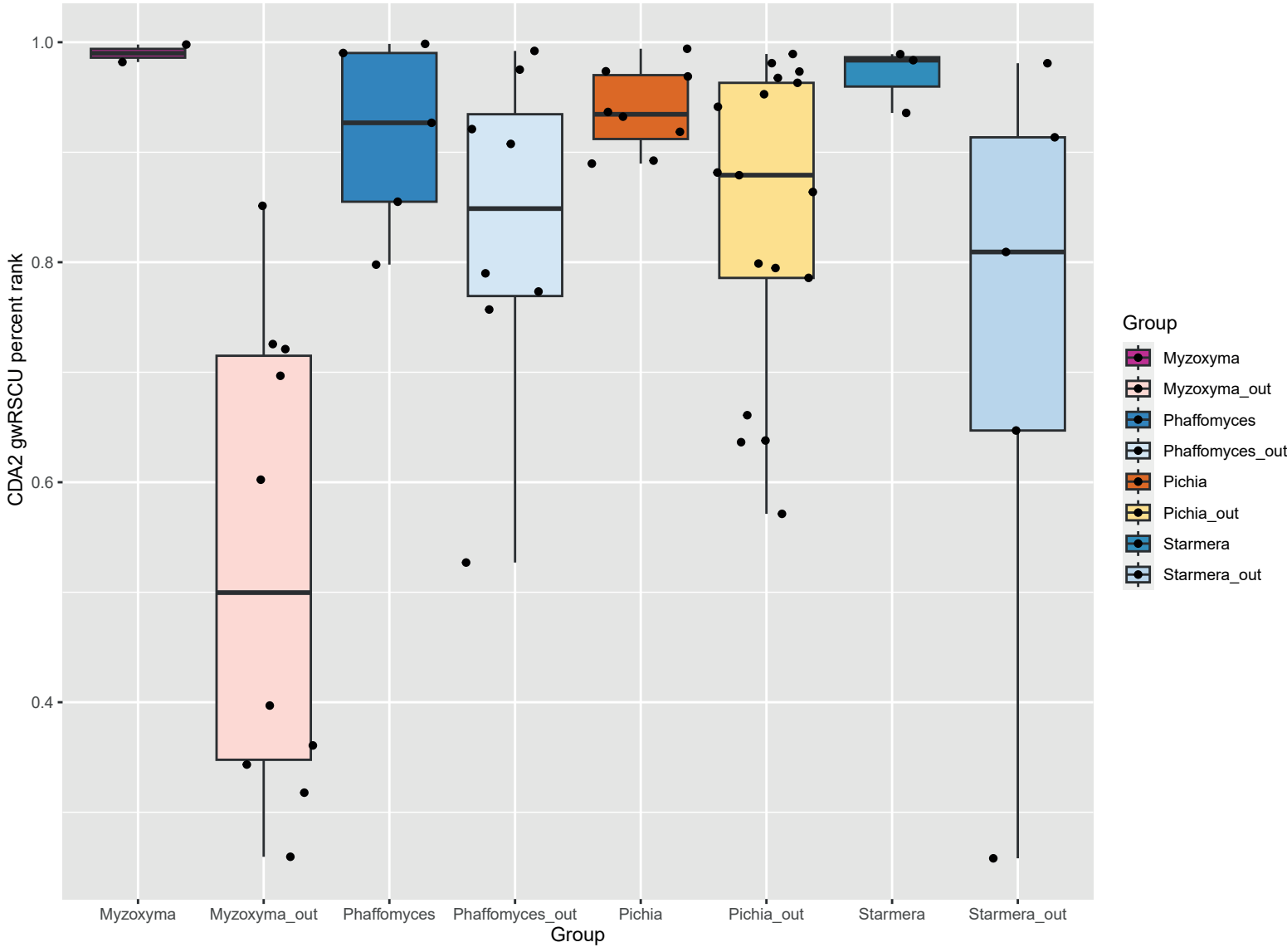

Supplement: S5 Fig — Cactophilic species belonging to Phaffomyces, Starmera, Pichia, and Myxozyma genera and their respective non-cactophilic closest relatives (identified as “out”) were inspected. The data underlying this Figure can be found in https://doi.org/10.6084/m9.figshare.24114381. (PDF) [file pbio.3002832.s005.pdf]

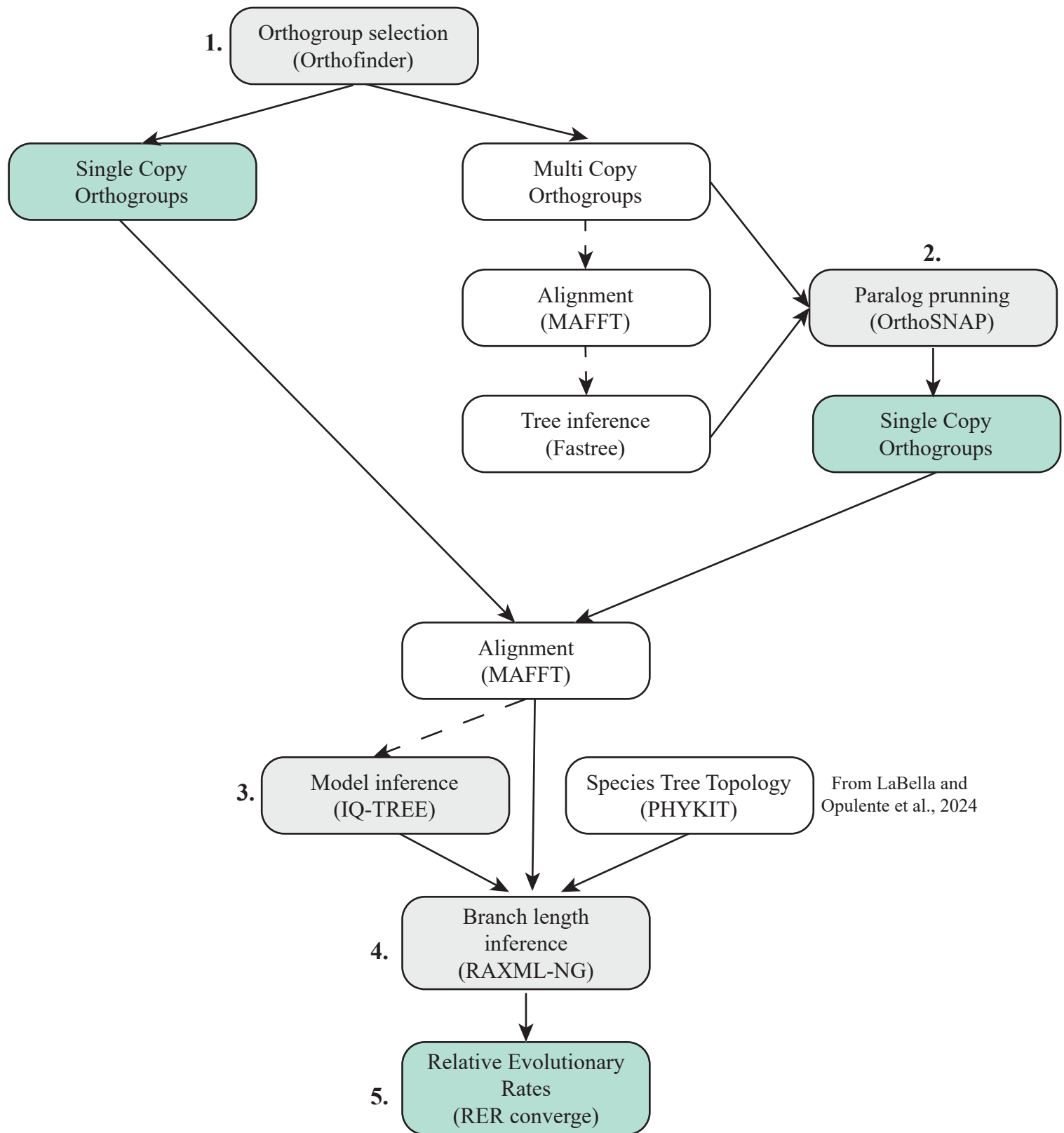

Supplement: S7 Fig — Schematic representation of the workflow for the detection of convergent evolutionary rates from orthogroup assignment and selection to RER converge analyses. (PDF) [file pbio.3002832.s007.pdf]
