# Supplementary material for: Diverse signatures of convergent evolution in cactus-associated yeasts
Source: PLoS Biol. 2024 Sep 23;22(9):e3002832. doi: 10.1371/journal.pbio.3002832 (PMC11449361; doi:10.1371/journal.pbio.3002832)

Only Metabolic
(strictly cactophilic)

Cross-validated Accuracy:
76.4%

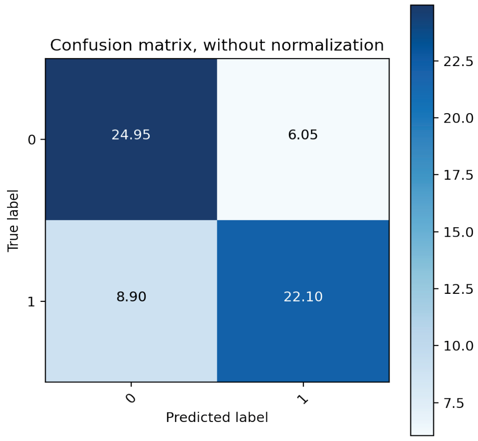

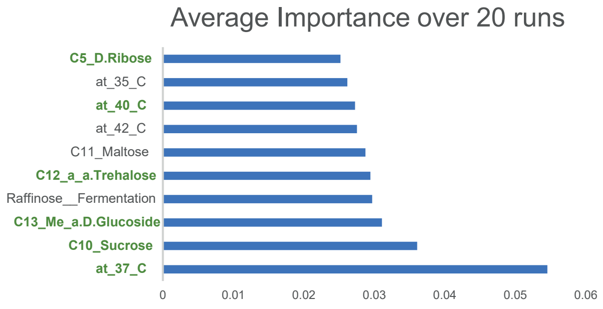

Only Genomic
(strictly cactophilic)

Cross-validated Accuracy:
72.2%

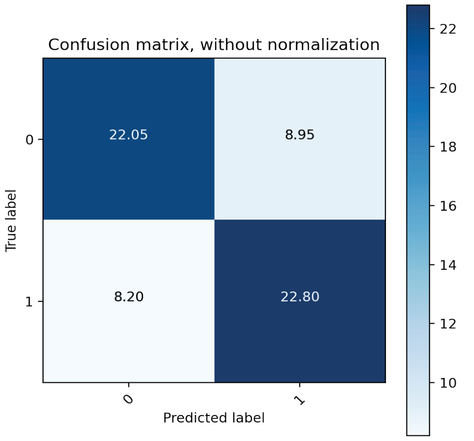

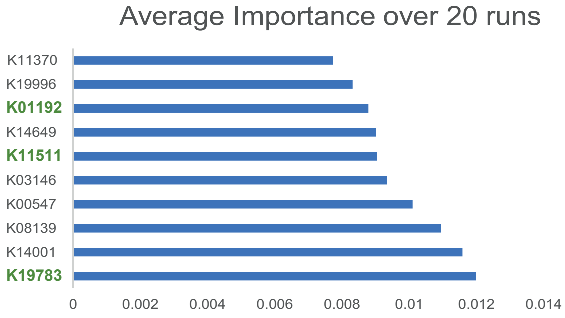

Only Metabolic
(strictly cactophilic
+ transient)

Cross-validated Accuracy:
73.0%

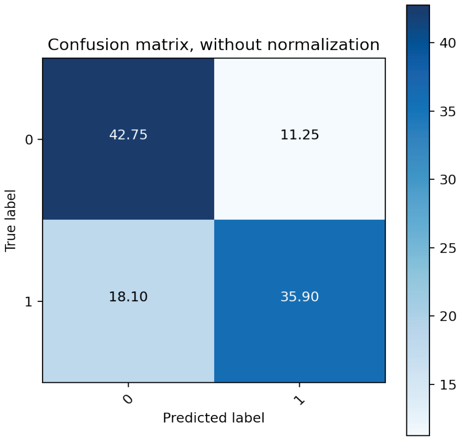

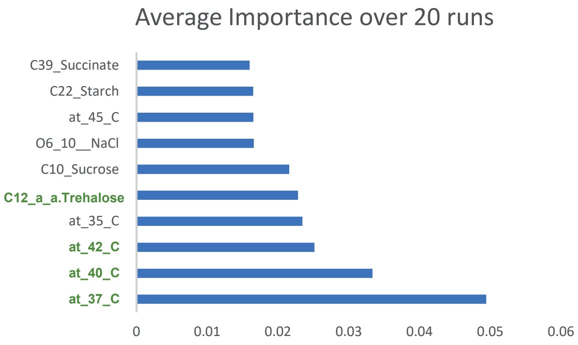

Only Genomic
(strictly cactophilic
+ transient)

Cross-validated Accuracy:
74.7%

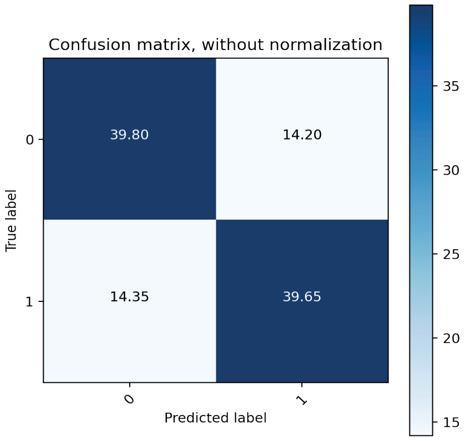

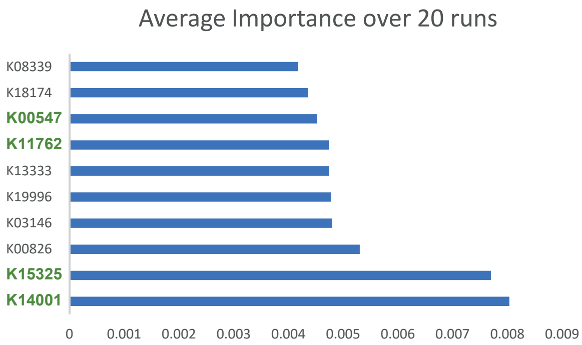

Supplement: S3 Fig — (A) Confusion matrices showing the average number of true positives, true negatives, false positives, and false negatives across strictly cactophilic and transient species resulting from 20 independent RF runs using either only metabolic or genomic data. On the right, the top 10 most important metabolic and genomic features for the RF classifier ranked according to their importance scores are shown. The features that overlap with the top 10 most important features resulting from the RF runs with both genomic and metabolic data (Fig 3) are highlighted in green. The data underlying this Figure can be found in S2 Table. (PDF) [file pbio.3002832.s003.pdf]
