# Supplementary material for: Diverse signatures of convergent evolution in cactus-associated yeasts
Source: PLoS Biol. 2024 Sep 23;22(9):e3002832. doi: 10.1371/journal.pbio.3002832 (PMC11449361; doi:10.1371/journal.pbio.3002832)
Supplement: S4 Fig — Branches selected for the analyses are shown (ω) in different colors (green for the foreground/cactophilic lineages and red for the background/non-cactophilic lineages). Only the genes for which no evidence of positive selection on the background lineages were considered for further analyses (please see Materials and methods section). (PDF) [file pbio.3002832.s004.pdf]

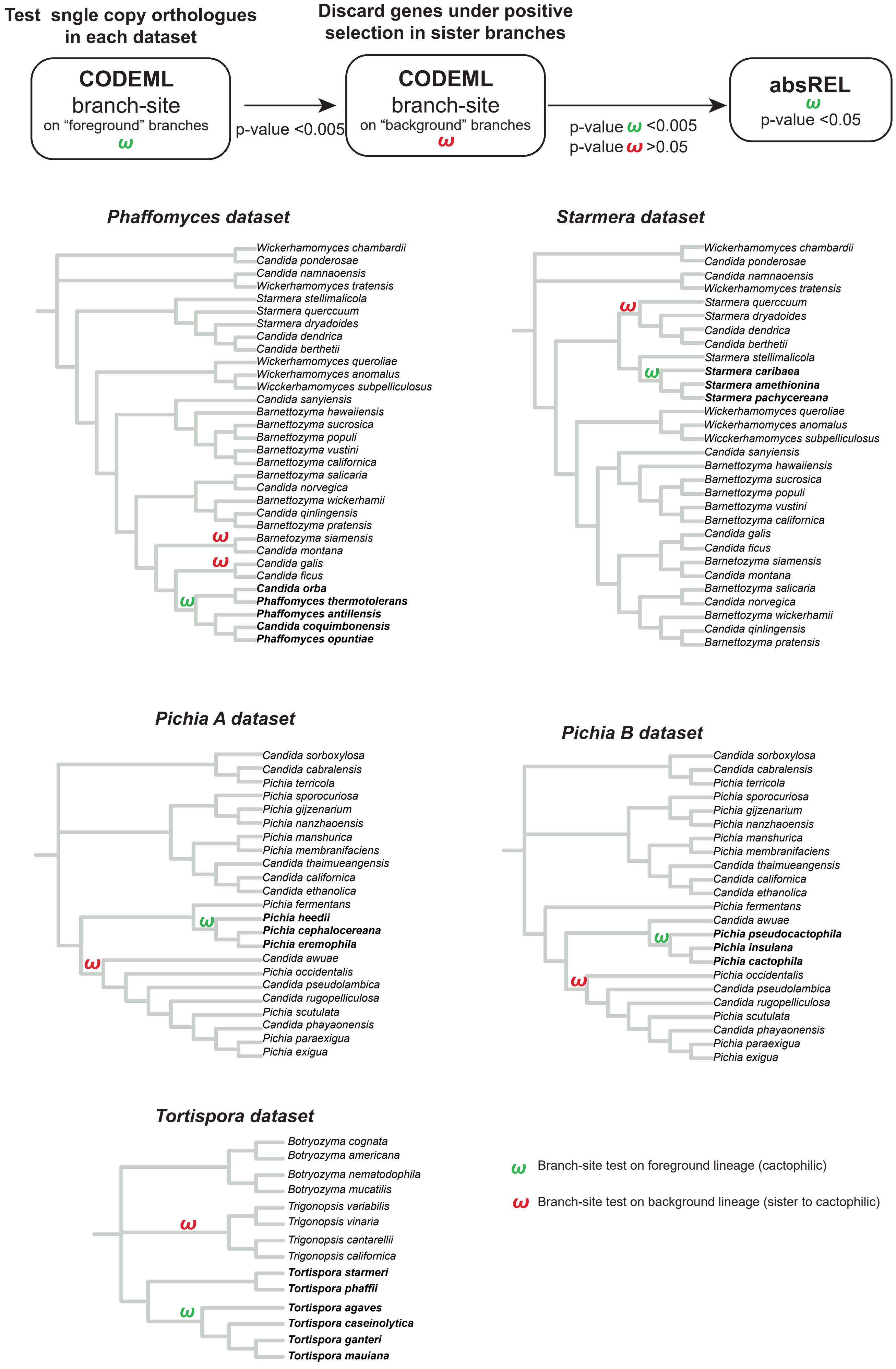
Test sngle copy orthologues in each dataset
CODEML branch-site on "foreground" branches ω
p-value <0.005
Discard genes under positive selection in sister branches
CODEML branch-site on "background" branches ω
p-value ω <0.005
p-value ω >0.05
absREL ω p-value <0.05
Phaffomyces dataset
Wickerhamomyces chambardii
Candida ponderosae
Candida namnaoensis
Wickerhamomyces tratensis
Starmera stellimalicola
Starmera querccuum
Starmera dryadoides
Candida dendrica
Candida berthetii
Wickerhamomyces queroliae
Wickerhamomyces anomalus
Wicckerhamomyces subpelliculosus
Candida sanyiensis
Barnettozyma hawaiiensis
Barnettozyma sucrosica
Barnettozyma populi
Barnettozyma vustini
Barnettozyma californica
Barnettozyma salicaria
Candida norvegica
Barnettozyma wickerhamii
Candida qinlingensis
Barnettozyma pratensis
Barnetozyma siamensis
Candida montana
Candida galis
Candida ficus
Candida orba
Phaffomyces thermotolerans
Phaffomyces antillensis
Candida coquimbonensis
Phaffomyces opuntiae
Starmera dataset
Wickerhamomyces chambardii
Candida ponderosae
Candida namnaoensis
Wickerhamomyces tratensis
Starmera querccuum
Starmera dryadoides
Candida dendrica
Candida berthetii
Starmera stellimalicola
Starmera caribaea
Starmera amethionina
Starmera pachycereana
Wickerhamomyces queroliae
Wickerhamomyces anomalus
Wicckerhamomyces subpelliculosus
Candida sanyiensis
Barnettozyma hawaiiensis
Barnettozyma sucrosica
Barnettozyma populi
Barnettozyma vustini
Barnettozyma californica
Candida galis
Candida ficus
Barnetozyma siamensis
Candida montana
Barnettozyma salicaria
Candida norvegica
Barnettozyma wickerhamii
Candida qinlingensis
Barnettozyma pratensis
Pichia A dataset
Candida sorboxylosa
Candida cabralensis
Pichia terricola
Pichia sporocuriosa
Pichia gijzenarium
Pichia nanzhaoensis
Pichia manshurica
Pichia membranifaciens
Candida thaimueangensis
Candida californica
Candida ethanolica
Pichia fermentans
Pichia heedii
Pichia cephalocereana
Pichia eremophila
Candida awuae
Pichia occidentalis
Candida pseudolambica
Candida rugopelliculosa
Pichia scutulata
Candida phayaonensis
Pichia paraexigua
Pichia exigua
Pichia B dataset
Candida sorboxylosa
Candida cabralensis
Pichia terricola
Pichia sporocuriosa
Pichia gijzenarium
Pichia nanzhaoensis
Pichia manshurica
Pichia membranifaciens
Candida thaimueangensis
Candida californica
Candida ethanolica
Pichia fermentans
Candida awuae
Pichia pseudocactophila
Pichia insulana
Pichia cactophila
Pichia occidentalis
Candida pseudolambica
Candida rugopelliculosa
Pichia scutulata
Candida phayaonensis
Pichia paraexigua
Pichia exigua
Tortispora dataset
Botryozyma cognata
Botryozyma americana
Botryozyma nematodophila
Botryozyma mucatilis
Trigonopsis variabilis
Trigonopsis vinaria
Trigonopsis cantarellii
Trigonopsis californica
Tortispora starmeri
Tortispora phaffii
Tortispora agaves
Tortispora caseinolytica
Tortispora ganteri
Tortispora mauiana
ω Branch-site test on foreground lineage (cactophilic)
ω Branch-site test on background lineage (sister to cactophilic)
