# Supplementary material for: Diverse signatures of convergent evolution in cactus-associated yeasts
Source: PLoS Biol. 2024 Sep 23;22(9):e3002832. doi: 10.1371/journal.pbio.3002832 (PMC11449361; doi:10.1371/journal.pbio.3002832)
Supplement: S6 Fig — Species that have been isolated from clinical contexts, and are emerging opportunistic pathogens, are highlighted. The ecological information presented was obtained from the CBS database and available literature according to the substrate of isolation of the type strain. (PDF) [file pbio.3002832.s006.pdf]

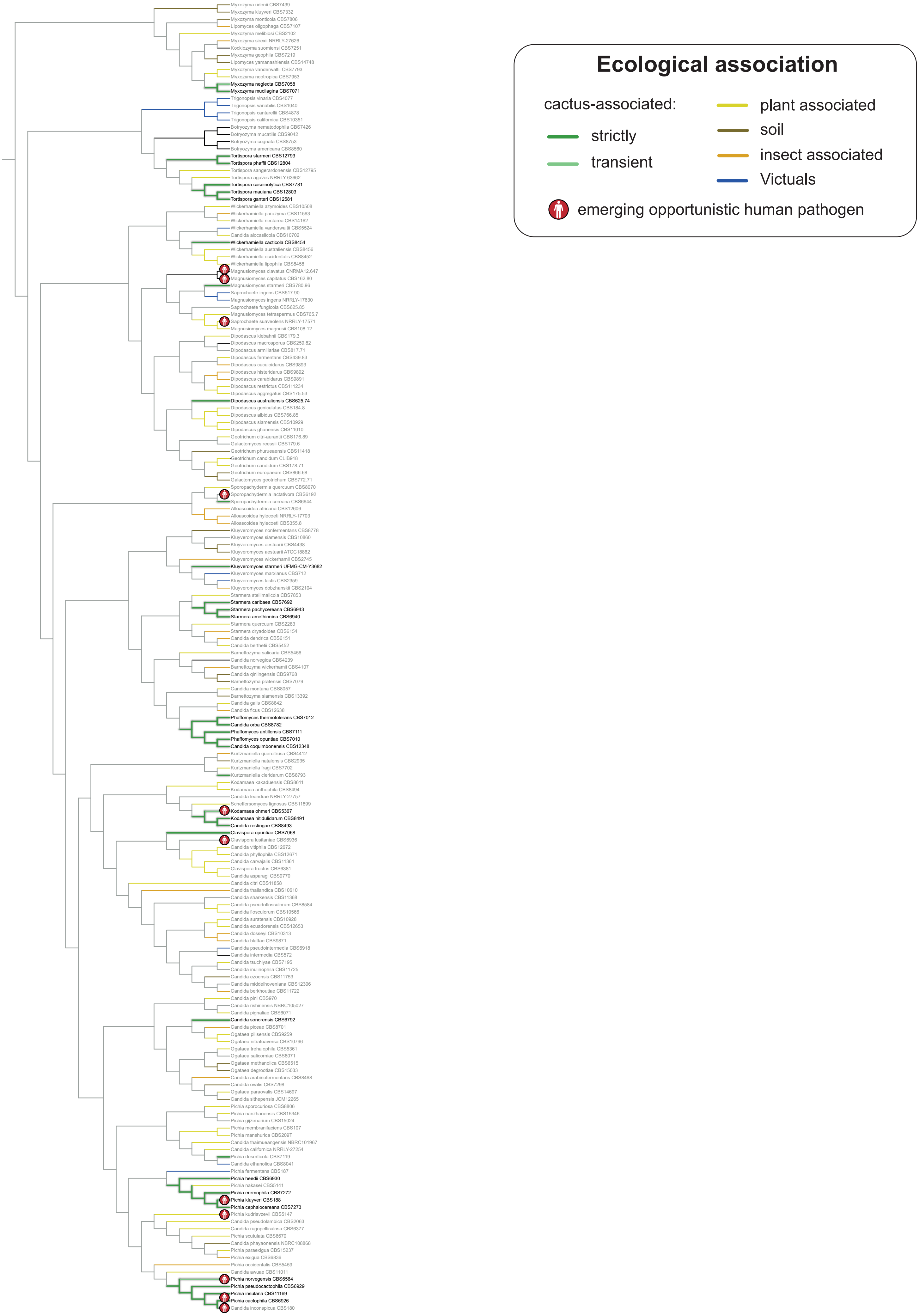
Ecological association
cactus-associated:
strictly
transient
plant associated
soil
insect associated
Victuals
emerging opportunistic human pathogen
Myxozyma udenii CBS7439
Myxozyma kluyveri CBS7332
Myxozyma monticola CBS7806
Lipomyces oligophaga CBS7107
Myxozyma melibiosi CBS2102
Myxozyma sirexii NRRLY-27626
Kockiozyma suomiensi CBS7251
Myxozyma geophila CBS7219
Lipomyces yamanashiensis CBS14748
Myxozyma vanderwaltii CBS7793
Myxozyma neotropica CBS7953
Myxozyma neglecta CBS7058
Myxozyma mucilagina CBS7071
Trigonopsis vinaria CBS4077
Trigonopsis variabilis CBS1040
Trigonopsis cantarellii CBS4878
Trigonopsis californica CBS10351
Botryozyma nematodophila CBS7426
Botryozyma mucatilis CBS9042
Botryozyma cognata CBS8753
Botryozyma americana CBS8560
Tortispora starmeri CBS12793
Tortispora phaffii CBS12804
Tortispora sangerardonensis CBS12795
Tortispora agaves NRRLY-63662
Tortispora caseinolytica CBS7781
Tortispora mauiana CBS12803
Tortispora ganteri CBS12581
Wickerhamiella azymoides CBS10508
Wickerhamiella parazyma CBS11563
Wickerhamiella nectarea CBS14162
Wickerhamiella vanderwaltii CBS5524
Candida alocasiicola CBS10702
Wickerhamiella cacticola CBS8454
Wickerhamiella australiensis CBS8456
Wickerhamiella occidentalis CBS8452
Wickerhamiella lipophila CBS8458
Magnusiomyces clavatus CNRMA12.647
Magnusiomyces capitatus CBS162.80
Magnusiomyces starmeri CBS780.96
Saprochaete ingens CBS517.90
Magnusiomyces ingens NRRLY-17630
Saprochaete fungicola CBS625.85
Magnusiomyces tetraspermus CBS765.7
Saprochaete suaveolens NRRLY-17571
Magnusiomyces magnusii CBS108.12
Dipodascus klebahnii CBS179.3
Dipodascus macrosporus CBS259.82
Dipodascus armillariae CBS817.71
Dipodascus fermentans CBS439.83
Dipodascus cucujoidarus CBS9893
Dipodascus histeridarus CBS9892
Dipodascus carabidarus CBS9891
Dipodascus restrictus CBS111234
Dipodascus aggregatus CBS175.53
Dipodascus australiensis CBS625.74
Dipodascus geniculatus CBS184.8
Dipodascus albidus CBS766.85
Dipodascus siamensis CBS10929
Dipodascus ghanensis CBS11010
Geotrichum citri-aurantii CBS176.89
Galactomyces reessii CBS179.6
Geotrichum phurueaensis CBS11418
Geotrichum candidum CLIB918
Geotrichum candidum CBS178.71
Geotrichum europaeum CBS866.68
Galactomyces geotrichum CBS772.71
Sporopachydermia quercuum CBS8070
Sporopachydermia lactativora CBS6192
Sporopachydermia cereana CBS6644
Alloascoidea africana CBS12606
Alloascoidea hylecoeti NRRLY-17703
Alloascoidea hylecoeti CBS355.8
Kluyveromyces nonfermentans CBS8778
Kluyveromyces siamensis CBS10860
Kluyveromyces aestuarii CBS4438
Kluyveromyces aestuarii ATCC18862
Kluyveromyces wickerhamii CBS2745
Kluyveromyces starmeri UFMG-CM-Y3682
Kluyveromyces marxianus CBS712
Kluyveromyces lactis CBS2359
Kluyveromyces dobzhanskii CBS2104
Starmera stellimalicola CBS7853
Starmera caribaea CBS7692
Starmera pachycereana CBS6943
Starmera amethionina CBS6940
Starmera quercuum CBS2283
Starmera dryadoides CBS6154
Candida dendrica CBS6151
Candida berthetii CBS5452
Barnettozyma salicaria CBS5456
Candida norvegica CBS4239
Barnettozyma wickerhamii CBS4107
Candida qinlingensis CBS9768
Barnettozyma pratensis CBS7079
Candida montana CBS8057
Barnettozyma siamensis CBS13392
Candida galis CBS8842
Candida ficus CBS12638
Phaffomyces thermotolerans CBS7012
Candida orba CBS8782
Phaffomyces antillensis CBS7111
Phaffomyces opuntiae CBS7010
Candida coquimbonensis CBS12348
Kurtzmaniella quercitrusa CBS4412
Kurtzmaniella natalensis CBS2935
Kurtzmaniella fragi CBS7702
Kurtzmaniella cleridarum CBS8793
Kodamaea kakaduensis CBS8611
Kodamaea anthophila CBS8494
Candida leandrae NRRLY-27757
Scheffersomyces lignosus CBS11899
Kodamaea ohmeri CBS5367
Kodamaea nitidulidarum CBS8491
Candida restingae CBS8493
Clavispora opuntiae CBS7068
Clavispora lusitaniae CBS6936
Candida vitiphila CBS12672
Candida phyllophila CBS12671
Candida carvajalis CBS11361
Clavispora fructus CBS6381
Candida asparagi CBS9770
Candida citri CBS11858
Candida thailandica CBS10610
Candida sharkensis CBS11368
Candida pseudoflosculorum CBS8584
Candida flosculorum CBS10566
Candida suratensis CBS10928
Candida ecuadorensis CBS12653
Candida dosseyi CBS10313
Candida blattae CBS9871
Candida pseudointermedia CBS6918
Candida intermedia CBS572
Candida tsuchiyae CBS7195
Candida inulinophila CBS11725
Candida ezoensis CBS11753
Candida middelhoveniana CBS12306
Candida berkhoutiae CBS11722
Candida pini CBS970
Candida rishiriensis NBRC105027
Candida pignaliae CBS6071
Candida sonorensis CBS6792
Candida piceae CBS8701
Ogataea pilisensis CBS9259
Ogataea nitratoaversa CBS10796
Ogataea trehalophila CBS5361
Ogataea salicorniae CBS8071
Ogataea methanolica CBS6515
Ogataea degrootiae CBS15033
Candida arabinofermentans CBS8468
Candida ovalis CBS7298
Ogataea paraovalis CBS14697
Candida sithepensis JCM12265
Pichia sporocuriosa CBS8806
Pichia nanzhaoensis CBS15346
Pichia gijzenarium CBS15024
Pichia membranifaciens CBS107
Pichia manshurica CBS209T
Candida thaimueangensis NBRC101967
Candida californica NRRLY-27254
Pichia deserticola CBS7119
Candida ethanolica CBS8041
Pichia fermentans CBS187
Pichia heedii CBS6930
Pichia nakasei CBS5141
Pichia eremophila CBS7272
Pichia kluyveri CBS188
Pichia cephalocereana CBS7273
Pichia kudriavzevii CBS5147
Candida pseudolambica CBS2063
Candida rugopelliculosa CBS6377
Pichia scutulata CBS6670
Candida phayaonensis NBRC108868
Pichia paraexigua CBS15237
Pichia exigua CBS6836
Pichia occidentalis CBS5459
Candida awuae CBS11011
Pichia norvegensis CBS6564
Pichia pseudocactophila CBS6929
Pichia insulana CBS11169
Pichia cactophila CBS6926
Candida inconspicua CBS180
