# Supplementary material for: Diverse signatures of convergent evolution in cactus-associated yeasts
Source: PLoS Biol. 2024 Sep 23;22(9):e3002832. doi: 10.1371/journal.pbio.3002832 (PMC11449361; doi:10.1371/journal.pbio.3002832)
Supplement: Attachment — Submitted filename: Response_reviewers_PLosBio_revised.docx [file pbio.3002832.s017.docx]

***Reviewer #1 (Remarks to the Author):***
 *I find most responses to be good and that the paper continues to be strong; however, there are 2 critical issues remaining:

1) In response to the rewording of what an accelerated evolution rate means, it is still incorrect. When an RER value is positive for a given branch, it reflects that the rate of evolution was faster on that branch compared to the rest of the branches for the same gene. It does not show that it is “faster than the average gene”, as written in the manuscript. The sign of an RER value has no relationship to the rates of other genes.*

We thank the reviewer for pointing out that our description of relative evolutionary rates of genes analyzed using RERconverge was incorrect – we have now fixed the issue throughout the manuscript.

*2) The issue of controlling for multiple tests for positive selection across genes must be corrected. When making thousands of tests, applying a cutoff to the nominal p-value is just not rigorous enough. It leaves the possibility that a large proportion of inferences (possibly all) could be false positives. (In this case, the proportion of genes falling below an alpha of 5% is less than 5% itself. Perhaps some observations are stronger than others, but without the p-values it cannot be judged.) Tests for selection on a single branch inherently lack power, especially on short branches. That is the reality of branch-site tests, and the source of the frustration of Ziheng Yang and Sergei Pond in the chat boards referenced by the authors.

This reviewer consulted with two experts about the best practice in general, without reference to this specific study. They uniformly required some way of controlling for multiple tests across genes. In Sergei Pond’s own recent papers, his group uses a false discovery rate, which has the advantage of allowing the user to control to a degree that is appropriate for the final use of the results. For example, in this study, a relatively high FDR would be acceptable (well above 5%) because the authors wish to make general statements about classes of positively selected genes in cactophillic lineages, rather than statements about specific genes. FDRs are a good compromise between other corrections that can be unnecessarily harsh (like Bonferroni) and not correcting at all.

In the end, I find this study excellent, but for full recommendation it should adhere to field best practices and a correction should be made.*

We thank the reviewers for further explaining their rationale on why we should perform multiple test corrections. We absolutely understand the Reviewers’ rationale, so we have now additionally performed multiple test corrections of the p-values obtained with CODEML using the Benjamini and Hochberg's FDR correction as suggested. First, we ran CODEML for the entire set of genes for all the five datasets, considering cactophilic clades as the foreground branches. We subsequently corrected those p-values using the Benjamini-Hochberg (BH) procedure and only retained those genes for which a corrected p-value was lower than 0.005. We performed a second round of corrections to exclude genes that also showed evidence of positive selection in background branches (i.e., genes with evidence of selection in non-cactophilic relatives). For that we performed branch-site tests in CODEML in the same way but considering the sister clade as the foreground branch (please see Fig. S4); genes for which a marginally significant signal for positive selection was obtained for the background clade (uncorrected p-value <0.05) were excluded. In this way, a conservative approach was employed, only considering genes for which a strong signal for selection was found specifically in cactophilic taxa but not in their non-cactophilic relatives. Finally, per the Reviewers’ earlier suggestions, we further validated genes inferred (by CODEML) to be under selection specifically in cactophilic taxa using the adaptive branch-site random effects likelihood test (aBSREL) implemented in HYPHY; genes that did not show evidence of selection with aBSREL were discarded.

In summary, genes considered to be candidates under selection *specifically* in cactophilic taxa in our study had to pass three criteria:

i) their BH-corrected p-value in the foreground branch was lower than 0.005,
ii) their uncorrected p-value in background branches was higher than 0.05, and
iii) their uncorrected p-value of foreground branches in aBSREL was lower than 0.05.

We have now included a new Table (Table S9) that shows the p-values before and after BH correction. We found that application of BH correction did not change the set of genes with strongest signatures of selection in our manuscript. This is because our original analyses also used a stricter p-value for genes with evidence of selection in cactophilic lineages (i.e., only the strongest candidates with evidence of selection in cactophilic taxa were retained) and a more lenient p-value for genes with evidence of selection in background lineages (i.e., any genes with weak evidence of selection in non-cactophilic taxa were excluded). While not formally correcting for multiple testing, this approach was conservative and removed nearly all genes removed by formal application of BH correction. Therefore, our interpretations and conclusions in this new version of the manuscript did not substantively change.

***Reviewer #2 (Remarks to the Author):***
 *I appreciate the author's considerable efforts. However, it's regrettable that they did not adequately address my main concerns of limited evidence, precisely that the data do not sufficiently support the theories and ideas of abstract and introduction about cacti associated yeast.

As the authors use data from their previous paper [51], the novel contribution of the current work should be to give convincing and statistically supported evidence to their research questions of what makes cactophilic yeast specific and how it might have evolved.
However, predicting cactophilic yeast remains to be at low accuracy, especially when using genomics data. The identified markers do not show a high importance for classification (the standard measure of mean decrease in classification is not reported, I assume because it is very low).
Also, if classification and distinguishing between scenarios is not the purpose of this study (as given in the answers to reviewer 1 and 2), then please apply standard statistical tests to identify markers and provide the p-values.*

We apologize for the lack of clarity explaining the main aim of this work, which was to find fingerprints of adaptation and convergent evolution in yeasts associated with cacti. Considering the extensive phenotypic and genomic data generated in Opulente et al., 2024, we found machine learning to be a promising starting point. We further note that that we explicitly discuss why we might not be able to distinguish between the proposed scenarios using machine learning (or any other approach) alone. Instead, we opted to use a combined approach that complements machine learning with gene family, evolutionary rates, and positive selection analyses. In this version of the manuscript, we explain our rationale for distinguishing between scenarios using this combined approach in the methods section; in the discussion and results section we discuss which scenario(s) fits best the results obtained. Finally, we have applied the statistical corrections requested (see our response below).

*There is also no novel data supported evidence for the relation of thermotolerant features with human pathogenicity. The added example case of cactophilic Pichia cactophila being closely related to human pathogens is truly interesting and supports the idea that thermotolerant species may have an impact on human health. However, the idea is already discussed in [51] and given the huge yeast data set the purpose of the manuscript should be to provide additional statistical evidence to support this theory in the context of cacti associated yeast. However, sufficient support based on the data is missing.
Suppl. Fig S5 is basically a copy of Fig.5 from [51] in which now cacti-associated yeast are highlighted. But Fig S5 does not support a clear relation of cacti-associated yeast with human pathogens. And the general idea of thermotolerance in yeast as a prerequisite for human pathogenicity was already discussed in [51].*

We agree that the data are still limited to establish a statistically supported relationship between cactophily and pathogenicity. We also do not claim that there is any novelty in the association between thermotolerance and human pathogenicity in yeasts; that thermotolerance is necessary but not sufficient to explain human pathogenicity in fungi has been well established. For these reasons, as well as due to the fact that our study’s aim is not to examine the origins of human pathogenicity in yeasts, we did not conduct any formal analyses. We simply wish to descriptively highlight and discuss the potential link between cactophilic yeast-thermotolerance-pathogenicity, given not only our observations but recent studies on emerging thermotolerant yeast pathogens that cluster within cactophilic clades (doi:10.1101/2023.12.27.573426). We mention that the cacti environment and probably other environments might be selecting for thermotolerance, raising the hypothesis that these types of environments might be launching pads for human pathogenicity.

*Also, to cancel some of my concerns with the argument that it’s “not the goal of the study” is ridiculous, as the abstract and introduction motivate in large parts that the paper will cover the questions of predicting cactophily and the relation of thermotolerance in yeast and human pathogens. It remains that the paper does not fulfill what the abstract suggests, including missing statistical support.*

It was not our intention to dismiss the reviewer’s comments and we sincerely apologize for not being able to convey our message properly. We agree with the reviewer’s comment that the accuracy of our machine learning analyses is moderate and have explicitly stated this throughout our manuscript. We have also discussed how to best interpret this finding and how the moderate accuracy of our machine learning analyses provides support for our proposed scenario II (Convergent phenotypes through divergent genotypes). This is now thoroughly discussed throughout the entire manuscript.

We also absolutely understand the Reviewers’ rationale, so we have now additionally performed multiple test corrections of the p-values obtained with CODEML using the Benjamini and Hochberg's FDR correction as suggested. First, we ran CODEML for the entire set of genes for all the five datasets, considering cactophilic clades as the foreground branches. We subsequently corrected those p-values using the Benjamini-Hochberg (BH) procedure and only retained those genes for which a corrected p-value was lower than 0.005. We performed a second round of corrections to exclude genes that also showed evidence of positive selection in background branches (i.e., genes with evidence of selection in non-cactophilic relatives). For that we performed branch-site tests in CODEML in the same way but considering the sister clade as the foreground branch (please see Fig. S4); genes for which a marginally significant signal for positive selection was obtained for the background clade (uncorrected p-value <0.05) were excluded. In this way, a conservative approach was employed, only considering genes for which a strong signal for selection was found specifically in cactophilic taxa but not in their non-cactophilic relatives. Finally, per the Reviewers’ earlier suggestions, we further validated genes inferred (by CODEML) to be under selection specifically in cactophilic taxa using the adaptive branch-site random effects likelihood test (aBSREL) implemented in HYPHY; genes that did not show evidence of selection with aBSREL were discarded.

In summary, genes considered to be candidates under selection *specifically* in cactophilic taxa in our study had to pass three criteria:

i) their BH-corrected p-value in the foreground branch was lower than 0.005,
ii) their uncorrected p-value in background branches was higher than 0.05, and
iii) their uncorrected p-value of foreground branches in aBSREL was lower than 0.05.

We have now included a new Table (Table S9) that shows the p-values before and after BH correction. We found that application of BH correction did not change the set of genes with strongest signatures of selection in our manuscript. This is because our original analyses also used a stricter p-value for genes with evidence of selection in cactophilic lineages (i.e., only the strongest candidates with evidence of selection in cactophilic taxa were retained) and a more lenient p-value for genes with evidence of selection in background lineages (i.e., any genes with weak evidence of selection in non-cactophilic taxa were excluded). While not formally correcting for multiple testing, this approach was conservative and removed nearly all genes removed by formal application of BH correction. Therefore, our interpretations and conclusions in this new version of the manuscript did not substantively change.

Overall, we think that our study is novel because it uses an unprecedented amount of genomic and phenotypic data to examine the repeated evolution of yeast cactophily. In our opinion, the fact that these data were produced and published to address different questions by Opulente et al., 2024 does not reduce the novelty of our work. While Opulente et al. examined the genetic bases of nutritional generalism and specialism, our study instead investigates the repeated evolution of a very interesting ecological trait, cactophily. We employed both similar (machine learning) but also distinct approaches (gene family analyses, evolutionary rates analyses, codon optimization analyses). We believe and anticipate that the data generated in Opulente et al., 2024 will continue to fuel more novel studies addressing a multitude of intriguing questions about yeast evolution.

***Reviewer #3 (Remarks to the Author):***
 *The authors have significantly improved the manuscript in response to the first round of reviews. I note that my comment on the calculation of the precision values was incorrect; the authors had the correct calculations (my mistake resulted from reading the confusion matrix in the wrong direction, the true values being on the y-axis).
Moreover, I find the justification for not correcting for multiple tests unsatisfying (in the rebuttal and the manuscript). I agree with the authors that correction for multiple testing is not required when only one branch is tested per gene. More precisely, the question "is gene X under positive selection?" can be answered without correcting the p-value if only one branch is tested. However, the question "how many genes are under positive selection" requires such a correction, whether the gene datasets are independent or not. For instance, Benjamini and Hochberg's FDR correction assumes that the tests are independent (but it is largely robust if they are not). So, stating that x genes are under positive selection with a nominal p-value of < 0.05 is ambiguous, as the type I error is - in such case - larger than 5%. This is the point made by Sergei Kosakovsky Pond in his comment referred to by the authors in their rebuttal. Ziheng Yang is not saying anything different: when the goal is to find candidate genes, ranking genes according to p-values is sufficient. But again, the issue of multiple testing arises when attempting to quantify how many genes may be under positive selection. As this quantification is not the main point here, I suggest that the authors adjust their conclusions instead of arguing against the use of an FDR procedure. For Tortispora, 2384 genes are tested and 31 are significant, which is significantly less than expected by chance alone (expected average: 119 genes). In Pichia, 261 genes are significant out of 2175, which is significantly higher than expected by chance (p-value 4e-38, expected average of 109 genes). In the other reported species, the numbers of significant genes are, unfortunately, in the range of what is expected by chance for the nominal p-value of 5%. This does not imply that the reported genes are not interesting candidates per se, but indicates no evidence for positive selection generally associated with cactophily transition. As a result, it may not be surprising that there is no overlap between candidate genes in all species, as this is expected if such gene sets are unrelated or random. I recommend that the authors convey this message more explicitly before examining further the candidate genes.*

We thank the reviewers for further explaining their rationale on why we should perform multiple test corrections. We absolutely understand the Reviewers’ rationale, so we have now additionally performed multiple test corrections of the p-values obtained with CODEML using the Benjamini and Hochberg's FDR correction as suggested. First, we ran CODEML for the entire set of genes for all the five datasets, considering cactophilic clades as the foreground branches. We subsequently corrected those p-values using the Benjamini-Hochberg (BH) procedure and only retained those genes for which a corrected p-value was lower than 0.005. We performed a second round of corrections to exclude genes that also showed evidence of positive selection in background branches (i.e., genes with evidence of selection in non-cactophilic relatives). For that we performed branch-site tests in CODEML in the same way but considering the sister clade as the foreground branch (please see Fig. S4); genes for which a marginally significant signal for positive selection was obtained for the background clade (uncorrected p-value <0.05) were excluded. In this way, a conservative approach was employed, only considering genes for which a strong signal for selection was found specifically in cactophilic taxa but not in their non-cactophilic relatives. Finally, per the Reviewers’ earlier suggestions, we further validated genes inferred (by CODEML) to be under selection specifically in cactophilic taxa using the adaptive branch-site random effects likelihood test (aBSREL) implemented in HYPHY; genes that did not show evidence of selection with aBSREL were discarded.

In summary, genes considered to be candidates under selection *specifically* in cactophilic taxa in our study had to pass three criteria:

iv) their BH-corrected p-value in the foreground branch was lower than 0.005,
v) their uncorrected p-value in background branches was higher than 0.05, and
vi) their uncorrected p-value of foreground branches in aBSREL was lower than 0.05.

We have now included a new Table (Table S9) that shows the p-values before and after BH correction. We found that application of BH correction did not change the set of genes with strongest signatures of selection in our manuscript. This is because our original analyses also used a stricter p-value for genes with evidence of selection in cactophilic lineages (i.e., only the strongest candidates with evidence of selection in cactophilic taxa were retained) and a more lenient p-value for genes with evidence of selection in background lineages (i.e., any genes with weak evidence of selection in non-cactophilic taxa were excluded). While not formally correcting for multiple testing, this approach was conservative and removed nearly all genes removed by formal application of BH correction. Therefore, our interpretations and conclusions in this new version of the manuscript did not substantively change.
