# Supplementary material for: Diverse signatures of convergent evolution in cactus-associated yeasts
Source: PLoS Biol. 2024 Sep 23;22(9):e3002832. doi: 10.1371/journal.pbio.3002832 (PMC11449361; doi:10.1371/journal.pbio.3002832)
Supplement: Attachment — Submitted filename: Response_reviewers_PLosBio_revision_v3.docx [file pbio.3002832.s019.docx]

*Reviewer #1:

As stated in my previous reviews of this manuscript, I am positive about this study. I found both the questions and datasets gathered by the authors to answer them innovative and of broad interest. The statistical issue of multiple testing, raised by all reviewers, has been given extra consideration in this revision, which addresses my last concern.

Minor:
- Line 422, "These tests were performed separately for each of the five cactophilic subclades": does this also imply that the BH correction was done for each clade independently, or was it conducted using all genes of all five clades simultaneously? (Both approaches can be justified; the precise methodology must be stated for clarity.)*

We thank the reviewer for the positive comments and for pointing out the missing information about the BH correction. The correction was done for each clade separately, because PAML calculations were also performed for each clade independently. This information is now included in the methods section (line 777, document with track changes).
